# Skin Autofluorescence, a Noninvasive Biomarker for Advanced Glycation End‐Products, Is Associated With Prevalent Vertebral and Major Osteoporotic Fractures: The Rotterdam Study

**DOI:** 10.1002/jbmr.4096

**Published:** 2020-06-22

**Authors:** Komal Waqas, Jinluan Chen, Fjorda Koromani, Katerina Trajanoska, Bram CJ van der Eerden, André G Uitterlinden, Fernando Rivadeneira, M Carola Zillikens

**Affiliations:** ^1^ Department of Internal Medicine Erasmus University Medical Center Rotterdam The Netherlands; ^2^ Department of Epidemiology Erasmus University Medical Center Rotterdam The Netherlands

**Keywords:** ADVANCED GLYCATION END PRODUCTS, BONE MINERAL DENSITY, OSTEOPOROSIS, PREVALENT FRACTURES, SKIN AUTOFLUORESCENCE

## Abstract

Advanced glycation end‐products (AGEs), which bind to type 1 collagen in bone and skin, have been implicated in reduced bone quality. The AGE reader™ measures skin autofluorescence (SAF), which might be regarded as a marker of long‐term accumulation of AGEs in tissues. We investigated the association of SAF with bone mineral density (BMD) and fractures in the general population. We studied 2853 individuals from the Rotterdam Study with available SAF measurements (median age, 74.1 years) and with data on prevalent major osteoporotic (MOFs: hip, humerus, wrist, clinical vertebral) and vertebral fractures (VFs: clinical + radiographic Genant’s grade 2 and 3). Radiographs were assessed 4 to 5 years before SAF. Multivariate regression models were performed adjusted for age, sex, BMI, creatinine, smoking status, and presence of diabetes and additionally for BMD with interaction terms to test for effect modification. Prevalence of MOFs was 8.5% and of VFs 7%. SAF had a curvilinear association with prevalent MOFs and VFs and therefore, age‐adjusted, sex stratified SAF quartiles were used. The odds ratio (OR) (95% confidence interval [CI]) of the second, third and fourth quartiles of SAF for MOFs were as follows: OR 1.60 (95% CI, 1.08–2.35; *p* = .02); OR 1.30 (95% CI, 0.89–1.97; *p* = .20), and OR 1.40 (95% CI, 0.95–2.10; *p* = .09), respectively, with first (lowest) quartile as reference. For VFs the ORs were as follows: OR 1.69 (95% CI, 1.08–2.64; *p* = .02), OR 1.74(95% CI, 1.11–2.71; *p* = .01), and OR 1.73 (95% CI, 1.12–2.73; *p* = .02) for second, third, and fourth quartiles, respectively. When comparing the top three quartiles combined with the first quartile, the OR (95% CI) for MOFs was 1.43 (95% CI, 1.04–2.00; *p* = .03) and for VFs was 1.72 (95% CI, 1.18–2.53; *p* = .005). Additional adjustment for BMD did not change the associations. In conclusion, there is evidence of presence of a threshold of skin AGEs below which there is distinctly lower prevalence of fractures. Longitudinal analyses are needed to confirm our cross‐sectional findings. © 2020 The Authors. *Journal of Bone and Mineral Research* published by American Society for Bone and Mineral Research.

## Introduction

Advanced glycation end‐products (AGEs) have been implicated in physiological mechanisms of aging and chronic diseases related to aging, such as cardiovascular diseases, Alzheimer’s disease, and complications of diabetes.^(^ ^1^, ^2^ ^)^ Although AGEs have been shown to accumulate in bone, the role of AGEs is not completely understood in osteoporosis, which is a major contributor to morbidity in the aging population. AGEs are a heterogeneous group of compounds formed as a result of non‐enzymatic glycation of proteins.^(^ ^3^, ^4^ ^)^ One such protein is type 1 collagen, which constitutes approximately 90% of the bone matrix proteins.^(^ ^5^ ^)^ AGEs bind to collagen in bone and develop crosslinking (pentosidine, versperlysine) and non‐crosslinking (carboxymethyl lysine [CML], carboxyethyl lysine) modifications of collagen molecules.^(^ ^6^, ^7^ ^)^ Moreover, AGEs interact with the receptor for advanced glycation end product (RAGE) on bone cells to generate inflammatory and oxidative mediators via nuclear factor κ‐B (NF‐κB).^(^ ^8^, ^9^ ^)^ Through these mechanisms, AGEs have been implicated in impaired bone biomechanical properties and reduced bone quality, especially in subjects with diabetes, who have higher levels of AGEs in bone and increased fracture risk despite normal bone mineral density (BMD).^(^ ^10^, ^11^, ^12^, ^13^, ^14^, ^15^ ^)^

Previous studies have reported a relationship between AGEs measured in serum, urine, and bone and bone strength reduction using different study designs. in vitro models, using bone samples incubated in glycated versus non‐glycated environment, have demonstrated that non‐enzymatic glycation of collagen leads to reduced bone quality without altering the bone quantity.^(^ ^1^, ^12^ ^)^ Studies using bone tissue excised from hip fracture patients have shown higher levels of the AGE pentosidine than hip osteoarthritis patients.^(^ ^16^, ^17^ ^)^ Studies using serum and urine pentosidine have shown either positive or no associations with fractures in subjects without type 2 diabetes (non‐T2DM) and with type 2 diabetes (T2DM).^(^ ^18^, ^19^, ^20^ ^)^ Serum CML has been reported to positively predict incident hip fractures in two population based cohorts.^(^ ^21^, ^22^ ^)^ Last, studies using serum CML or pentosidine have shown no relation,^(^ ^21^ ^)^ or negative association, with BMD.^(^ ^23^, ^24^ ^)^ Hence, inconsistent outcomes have been observed depending on the type and localization of AGEs studied in association with the bone parameters.

Several techniques, such as mass spectrometry and high‐performance liquid chromatography, have been employed to measure AGEs in different body compartments, including serum, urine, and tissues. However, there is currently no standardized way to measure AGEs, and the extent to which serum or urine AGEs represent tissue AGEs is controversial.^(^ ^25^ ^)^ A promising technique is skin autofluorescence (SAF), which measures skin AGEs based on their fluorescent properties.^(^ ^26^, ^27^ ^)^ It is a very quick, user‐friendly, and highly reproducible technique.^(^ ^5^ ^)^ It is noteworthy that AGEs bind to type 1 collagen in both skin and bone.^(^ ^5^ ^)^ Studies using human cadavers have shown that skin and bone pentosidine levels per milligram of collagen are correlated with each other.^(^ ^28^ ^)^ Additionally, skin AGEs measured by SAF have been associated with reduced bone material strength index (BMSi) measured with reference point indentation, a potential measure of bone quality, in a small cross‐sectional study in postmenopausal women with T2DM.^(^ ^29^ ^)^ Thus, measuring AGE levels by means of SAF might be a reflection of bone AGEs and a potential biomarker of bone strength and fracture risk.

The relationship of skin AGEs with BMD and fracture risk in the general population remains to be established. The aim of the present study is to investigate the associations between SAF, BMD, and prevalent fractures in a cohort of elderly people from the general population including individuals with diabetes from the Rotterdam study (RS).

## Subjects and Methods

### Study population

This cross‐sectional analysis is a part of the RS, an ongoing population‐based, prospective cohort study. The RS was set up in 1990 in the Ommoord district of the city Rotterdam. The study population consists of three cohorts that were included in three different periods: RS‐I in 1990, RS‐II in 2000, and RS‐III in 2006. The inhabitants above 55 years of age in RS‐I and RS‐II and above 45 years of age in RS‐III were invited for inclusion. All participants were examined at baseline and every 3 to 5 years at follow‐up examinations. The design and objectives of the study have been extensively described.^(^ ^30^ ^)^ The RS was approved by the institutional review board (Medical Ethics Committee) of Erasmus Medical Center. All participants in the present analysis provided written informed consent to participate.

The participants of the RS with SAF measurement constitute almost 40% of the total alive participants from that cycle. We included 2853 participants from RS‐I (6th follow‐up, *n* = 705 [25%]), RS‐II (4th follow‐up, *n* = 1034 [36%]), and RS‐III (2nd follow‐up, *n* = 1114 [39%]) with available SAF measurements. We excluded participants with missing data on informed consent, effective glomerular filtration rate (eGFR), BMI, smoking status, and diabetes status (Supplemental Fig. 1).

### Measurement of SAF

The AGE Reader™ (DiagnOptics Technologies B.V., Groningen, The Netherlands) was introduced in the RS in 2013 to measure SAF noninvasively. SAF value measured by the AGE Reader has a moderately strong correlation with the amount of AGEs measured in skin biopsies.^(^ ^26^, ^27^ ^)^ The device exploits the fluorescent properties of AGEs. Skin fluorescence has also been evaluated as a clinical tool for noninvasive assessment of AGEs in tissues and associated with long‐term complications of diabetes.^(^ ^31^, ^32^ ^)^

Participants were instructed not to apply any lotion or creams on the dominant forearm for 2 days prior to the measurement. The AGE Reader illuminates 4 cm^2^ of skin on the dominant forearm with an excitation light source with a peak wavelength at 370 nm.^(^ ^27^ ^)^ Emission light with the wavelength of 420 to 600 nm and reflected excitation light with a wavelength of 300 to 420 nm from the skin is measured by the AGE reader. AGE Reader software has been programmed to automatically estimate SAF based on the ratio of emitted and reflected light spectrums. Our AGE Reader, based on a validated algorithm, corrects for skin reflectance between 6% and 10% and excludes participants with a skin reflectance less than 6% (darker‐skinned individuals). Values were defined as outliers in SAF and excluded from the analysis if they exceeded the scope of mean + 4SD; based on this, eight participants were excluded.

### Prevalent major osteoporotic fractures

Fracture events that occurred after the age of 45 years for RS‐III and after the age of 55 years for RS‐I and RS‐II were included until the end of 2012. All fracture events were reported by general practitioners in the research area by means of computerized systems and by research physicians or trained nurses outside the research area or through hospital records. All events reported were verified by research physicians who independently reviewed and coded the information. Subsequently, a medical expert reviewed all inconsistencies in coded events for final classification.

Fractures were included if they are a component of major osteoporotic fractures (MOFs), which includes fracture of the hip, vertebra (clinical), wrist, or humerus. These fractures are the basis for the 10‐year absolute fracture risk estimates via the Fracture Risk Assessment Tool (FRAX) used in multiple large‐scale clinical studies.^(^ ^33^ ^)^ Clinical vertebral fractures were defined as those that came to medical attention when subjects with symptoms (mainly back pain) visited the medical practitioner and confirmation of fractures occurred on spine radiographs. All fractures were coded according to the International Classification of Diseases and Related Health Problems, 10th Revision (ICD‐10).

### Radiographic vertebral fractures

All thoracolumbar radiographs were obtained by a digitalized Fuji FCR system (FUJIFILM Medical Systems, Stanford, CA, USA) according to a standardized protocol described elsewhere.^(^ ^34^ ^)^ Radiographic vertebral assessment data was available for 2085 subjects with available SAF measurements till the end of 2008. Vertebral fractures were classified using vertebral morphometry grading 1 to 3 (OPTASIA‐Spina Analyzer; Optasia Medical, Cheadle, UK).^(^ ^35^ ^)^ Because there is doubt whether grade 1 (mild) deformities represent true osteoporotic vertebral fractures or not, we considered grade 2 (moderate) and grade 3 (severe) fractures as radiographic vertebral fractures.^(^ ^36^ ^)^

### Prevalent vertebral fractures

For this study, prevalent vertebral fractures (VFs) were defined as a combination of any vertebral fracture identified on either a radiograph as grade 2 (moderate) or grade 3 (severe) deformity or a clinically reported spine fracture. Therefore, we performed analyses on the most clinically relevant vertebral fractures.^(^ ^36^ ^)^

### Measurement of BMD

BMD was measured at the femoral neck (FN) and lumbar spine (LS) using iDXA Prodigy total body fan‐beam densitometer (GE Lunar Corp., Madison, WI, USA) as reported here.^(^ ^30^ ^)^ All scans were performed and verified by a trained technician who applied adjustments when necessary. Sex‐specific *T*‐scores for FN and LS‐BMD were calculated using the Third National Health and Nutrition Examination Survey (NHANES III) reference population.^(^ ^37^ ^)^ A total of 2654 participants (missing = 199) had complete data on FN‐BMD and LS‐BMD. The subjects from the cohort RS‐I (6th follow‐up, *n* = 624 or 24%) and RS‐II (4th follow‐up, *n* = 978 or 36%) underwent BMD scanning on average 4 to 5 years before SAF measurements. RS‐III (2nd follow‐up, *n* = 1052 or 40%) had cross‐sectional measurements of both FN‐BMD and LS‐BMD.

### Measurement of trabecular bone score

Trabecular bone score (TBS) was analyzed using TBS iNsight software (Med‐Imaps, Geneva, Switzerland). Briefly, TBS is a novel gray‐level texture measurement, extracted from DXA images, which correlates with 3D parameters of bone microarchitecture, connectivity density, trabecular separation, and trabecular number. For each region of measurement, TBS was evaluated based on gray‐level analysis of the DXA images as the slope at the origin of the log–log representation of the experimental variogram. The method of TBS assessment has been described in detail elsewhere.^(^ ^38^ ^)^

A total of 2654 participants had complete data on TBS derived from the same DXA images from which BMD was measured. We performed our analysis on 2583 participants after excluding those with a BMI above 37 as TBS calculations are not reliable in those obese subjects.

### Assessment of associated factors

Height (cm) and weight (kg) were measured in the research center with the individuals in standing position wearing indoor clothing without shoes. BMI was computed as weight in kilograms divided by height in meters squared (kg/m^2^). T2DM was defined by combining information on antidiabetic medication use, fasting blood glucose levels, or diagnosis in the GP registries. Smoking status was classified as current, ex‐ or never smokers collected through self‐report during home interviews.

### Biochemistry

Serum creatinine and serum fasting glucose were measured through automated enzymatic method. eGFR was calculated by the Chronic Kidney Disease Epidemiology Collaboration (CKD‐EPI) equation using serum creatinine concentration, age, and sex data.^(^ ^30^ ^)^

### Statistical methods

Statistical analyses were performed through IBM SPSS statistics 24 (version 24.0; IBM Corp., Armonk, NY, USA). Normality of data was determined by the use of histograms and Shapiro‐Wilk test. Depending on the normal or non‐normal distribution, data is presented as mean ± SD or median (interquartile range, IQR), respectively. Means of continuous variables among groups were compared via the use of Mann‐Whitney‐Wilcoxon test when a non‐normal distribution was assumed or independent samples *t* test or ANOVA when the variable was normally distributed. Chi‐square test was adopted to compare the means of categorical variables.

We identified potential confounders in the relationship between exposure (SAF) and outcome (BMD/TBS/fractures) based on literature evidence. Age, sex, BMI, eGFR, smoking status, diabetes status, and RS cohorts were included as covariates to study these relationships and consistently used in fully adjusted models. Linear regression analysis was used to assess the relationship between SAF and BMD at the LS and FN and SAF and TBS. In linear regression analysis, all models included a time variable taking into account the difference in years, if any, between SAF and BMD/TBS measurements. Logistic regression analysis was performed to assess whether SAF was associated with the presence of prevalent VFs or MOFs. A centered linear (cSAF) and polynomial term (cSAF^2^) was added to the logistic regression model to check for the curvilinear effects between SAF and fractures (Supplemental Table 1a and b). A comparison of linear versus polynomial model in studying the association of SAF and MOFs using Akaike information criterion (AIC) (1672.8 versus 1664.5) and −2log‐likelihood (−2LL) (1515.2 versus 1505.6) and for SAF and VFs with AIC (1384.2 versus 1375.0) and −2LL (1410.6 versus 1402.7) showed that a polynomial model provides a better fit to the data. Although a polynomial model fitted best to study this relation, we still studied SAF in quartiles to allow for a better comparison with the existing literature and for easier interpretation of our results.

Henceforth, sex‐stratified, age‐adjusted SAF scores were calculated for the total population, because SAF and fractures are strongly affect by age and sex.^(^ ^39^, ^40^ ^)^ Next, we classified individuals into quartiles of their age‐adjusted SAF scores, in men: first quartile (Q1) SAF ≤ −0.68, second quartile (Q2) −0.68 < SAF < −0.12, third quartile (Q3) −0.12 < SAF < 0.58, and highest quartile (Q4) SAF ≥0.58; in women: first quartile Q1 SAF ≤ −0.71, second quartile Q2 − 0.71 < SAF < −0.09, third quartile Q3 –0.09 < SAF < 0.59, and highest quartile Q4 SAF ≥0.59.

We tested for interaction terms between SAF and diabetes status, smoking status, eGFR, and sex in the multivariate fully adjusted models, and report results separately where statistically significant (*p* for interaction ≤ .10 for each) interaction was found. Because AGEs have been repeatedly implicated in pathogenesis of increased fracture risk in T2DM,^(^ ^13^ ^)^ we performed sensitivity analyses in participants with and without T2DM irrespective of statistical interaction.

## Results

### General characteristics

Table 1 shows baseline characteristics of study participants by quartiles of sex‐stratified, age‐adjusted SAF. There were generally no significant differences in baseline characteristics across quartiles of SAF (all *p* > .05) except that an increasing prevalence of T2DM subjects (*p* value for trend = .01) and current smokers (*p* value for trend = .05) were observed from the first to the fourth quartile. There was no significant increasing or decreasing trend for the bone parameters, namely FN‐BMD, LS‐BMD, or TBS, from the first to the fourth quartile. The prevalence of MOFs from the first quartile to the fourth quartile did not show an increasing trend (*p* value for trend = 0.27). The third quartile of SAF (Q3) had significantly lower number of MOFs than Q2 and Q4. The prevalence of VFs increased significantly from Q1 to Q4 (*p* value for trend = .02).

**Table 1:** Baseline Characteristics of Rotterdam Study Participants Categorized by Age‐Adjusted, Sex‐Stratified SAF Quartiles

	Quartile 1 (*n* = 714)	Quartile 2 (*n* = 713)	Quartile 3 (*n* = 713)	Quartile 4 (*n* = 713)	*p* ^a^
SAF (A.U.), mean ± SD	1.88 ± 0.23	2.18 ± 0.18	2.5 ± 0.19	3.0 ± 0.36	NA
Males, *n* (%)	310 (43)	309 (43)	309 (43)	310 (43)	NA
Age (years), median (IQR)	75.2 (14.2)	72.6 (15.3)	73.2 (14.1)	74.8 (13.0)	**<.001**
BMI (kg/m^2^), mean ± SD	27.2 ± 3.8	27.3 ± 4.1	27.8 ± 4.5	27.9 ± 4.8	**.03**
eGFR (mL/min), median (IQR)	78.9 (18.0)	80.5 (20.6)	78.8 (19.4)	78.0 (23.1)	**.05**
T2DM (%)	8.4	10.5	14.8	20.8	**<.001**
Smoking status (%)					**<.001**
Current	10	14	14	24	
Past	51.5	54	57	49	
Never	38.5	32	29	27	
FN‐BMD (g/cm^2^), mean ± SD	0.903 ± 0.134	0.907 ± 0.138	0.907 ± 0.136	0.892 ± 0.131	.10
FN‐BMD *T*‐score, mean ± SD	−1.19 ± 0.86	−1.17 ± 0.88	−1.17 ± 0.85	−1.27 ± 0.82	.12
LS‐BMD (g/cm^2^), mean ± SD	1.134 ± 0.206	1.113 ± 0.204	1.147 ± 0.196	1.144 ± 0.216	.45
LS BMD *T*‐score, mean ± SD	−0.43 ± 1.62	−.45 ± 1.60	−0.31 ± 1.58	−0.38 ± 1.67	.42
TBS, mean ± SD	1.312 ± 0.097	1.312 ± 0.097	1.310 ± 0.099	1.305 ± 0.103	.26
MOFs, *n* (%)	53 (7)	66 (9.5)	58 (9)	68 (9.5)	.27
VFs, *n* (%)	34 (5)	50 (7)	53 (7)	56 (8)	**.02**

a Values of *p* < .05 are considered significant and are shown in bold.

A.U. = arbitrary units; BMI = body mass index; FN‐BMD = femoral neck bone mineral density; LS‐BMD = lumbar spine bone mineral density; MOF = major osteoporotic fracture; NA = not applicable; SAF = skin autofluorescence; TBS = trabecular bone score; VF = vertebral fractures.

For the total study population, the median age was 74.1 years (IQR, 66.9 to 81.1 years) and the mean value of SAF was 2.33 ± 0.53 arbitrary units (A.U.). A total of 254 participants (8.5%) had a prevalent MOF at the time of SAF measurements including 80 clinical vertebral, 26 hip, 123 wrist, and 39 humerus fractures. A total of 193 participants (7%) had a prevalent VF at the time of analysis. A comparison of the basic demographics between T2DM and non‐T2DM subjects showed that participants with T2DM were older, had higher BMI, were more often males, and their SAF values were higher than participants without T2DM (2.57 ± 0.50 A.U. versus 2.36 ± 0.47 A.U.). A comparison of bone parameters showed that subjects with T2DM had higher BMD at both FN (0.922 ± 0.14 versus 0.899 ± 0.13, *p* = .003) and LS (1.18 ± 0.20 versus 1.13 ± 0.21, *p* < .001) than those without T2DM but no difference in prevalence of MOFs and VFs.

### Linear regression analysis of SAF with BMD and TBS

Table 2 shows the results of the linear regression analysis describing the association of SAF with FN‐BMD and LS‐BMD. There was a weak but statistically significant correlation coefficient between FN‐BMD and SAF (*r* = −0.033; *p* = .09) as well as between LS‐BMD and SAF (*r* = 0.096; *p* < .001) in an unadjusted model. This association attenuated after controlling for age and sex for FN‐BMD (β = −0.016; *p* = .39) as well as LS‐BMD (β = 0.037; *p* = .06) and totally disappeared in fully adjusted models (FN‐BMD: β = −0.021, *p* = .29; LS‐BMD: β = 0.014, *p* = .47).

**Table 2:** Association of SAF With Femoral Neck and Lumbar Spine BMD and TBS in the Whole Population and Stratified by Sex (*n* = 2654)

	Model 1		Model 2		Model 3	
Parameter	Standardized coefficient β	*p*	Standardized coefficient β	*p*	Standardized coefficient β	*p*
Femoral neck BMD^a^						
All	−0.033	.09	−0.016	.39	−0.021	.29
Females	−0.081	**.002**	−0.059	**.02**	−0.051	**.04**
Males	−0.022	.45	0.002	.83	0.004	.69
Lumbar spine BMD^a^						
All	0.096	**<.001**	0.037	.06	0.014	.47
Females	0.011	.68	0.022	.40	−0.005	.81
Males	0.054	.08	0.047	.15	0.026	.45
TBS						
All	−0.038	**.05**	−0.036	.07	−0.026	.19

Values are shown as standardized coefficient β per arbitrary unit and *p* value. Bold values are significant at *p* ≤ .05. Model 1 = unadjusted; model 2 = adjusted for age (and sex if non‐stratified by sex); model 3 = additionally adjusted for DM status, BMI, creatinine, smoking, RS cohorts, and time variable.

A.U. = arbitrary units; BMI = body mass index; FN‐BMD = femoral neck bone mineral density; LS‐BMD = lumbar spine bone mineral density; MOF = major osteoporotic fracture; NA = not applicable; SAF = skin autofluorescence; TBS = trabecular bone score; VF = vertebral fractures.

a Value of *p* for interaction for SAF*sex is 0.003 for FN‐BMD, 0.05 for LS‐BMD, and 0.55 for TBS; *p* < .10 is used for stratification.

In fully adjusted linear regression models, there was a difference by sex in the association between SAF and BMD (*p* value for interaction for SAF*sex for FN‐BMD: .003, and for LS‐BMD: .05). After stratification by sex, there was a marginally significant negative association of SAF with FN‐BMD only in females (β = −0.051; *p* = .04) in a fully adjusted model. On the other hand, there was no association between LS‐BMD and SAF in either of the sexes.

Table 2 also shows the results of linear regression analysis describing the association of SAF with TBS. In unadjusted model, the correlation coefficient was marginally significant (β = −0.38; *p* = .05). The association between SAF and TBS (β = −0.36; *p* = .07) was minimally attenuated after adjusting for age and sex, and totally disappeared after adjusting for eGFR, smoking, diabetes status, and BMI (β = −0.26; *p* = .19). There was no effect modification by sex or diabetes status (all *p* > .10).

### Logistic regression analysis for the prevalence of fractures across sex‐stratified, age‐adjusted SAF quartiles

**Fig 1:**
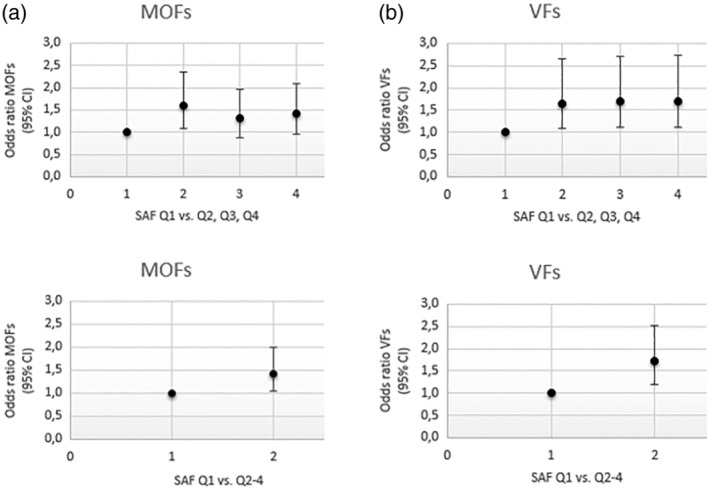
(*A*) Odds ratio for prevalent major osteoporotic fractures by sex‐stratified, age‐adjusted quartiles of SAF levels adjusted for age, sex, BMI, smoking, eGFR, RS cohorts, and diabetes status. (Note that SAF Q1 with the lowest value of SAF is the reference). (*B*) Odds ratio for prevalent radiological vertebral fractures by sex‐stratified, age‐adjusted quartiles of SAF levels adjusted for age, sex, BMI, smoking, eGFR, RS cohorts, and diabetes status. (Note that SAF quartile 1 with the lowest value of SAF is the reference). Q = quartile; SAF = skin autofluorescence.

At the time of SAF measurement, there were 245 participants with prevalent MOFs and 193 participants with prevalent VFs. The analysis was performed consistently including the sex stratified, age‐adjusted SAF quartiles as a predictor in every model, and fractures, either MOFs or VFs as outcome. Figure 1*A* and 1*B* shows the results of logistic regression describing the association between SAF quartiles and the presence of MOFs or VFs in fully adjusted models including age, sex, BMI, smoking, RS cohorts, diabetes status, and eGFR.

#### MOFs

In a fully adjusted model, the ORs (95% CI, *p* value) of the second, third, and fourth (highest) quartiles of SAF were 1.60 (95% CI, 1.08–2.35; *p* = .02), 1.33 (95% CI, 0.89–1.97; *p* = .16), and 1.41(95% CI, 0.95–2.10; *p* = .08), respectively, when the bottom quartile with lowest SAF values was used as reference (Fig. 1*A*). The corresponding ORs (95% CI; *p* value) of the top three quartiles combined for MOFs was 1.43 (95% CI, 1.04–2.00; *p* = .03) versus the bottom quartile of SAF (Fig. 1*B*).

#### Prevalent VFs

In a fully adjusted model, the ORs (95% CI; *p* value) of the second, third, and fourth (highest) quartile of SAF were 1.65 (95% CI, 1.08–2.65; *p* = .03), 1.70 (95% CI, 1.11–2.71; *p* = .02), and 1.70 (95% CI, 1.12–2.73; *p* = .02), respectively, when the bottom quartile was used as reference (Fig. 1*B*). The corresponding OR (95% CI; *p* value) of the top three quartiles combined for VFs was 1.73 (95% CI, 1.18–2.53; *p* = .005) versus the bottom quartile of SAF (Fig. 1*B*).

#### Logistic regression in subjects with available BMD measurements

FN‐BMD and LS‐BMD were available in a subset of 2654 participants. In logistic regression models for the subcohort with available BMD measurements, the association of higher SAF levels with MOFs was no more statistically significant due to loss of power (*n* = 199 missing BMD data), but the effect size did not change with or without addition of either FN‐BMD or LS‐BMD (Table 3). The association of higher SAF values with VFs was, however, not attenuated by the addition of either FN‐BMD or LS‐BMD, but was still statistically significant (Table 3).

**Table 3:** Odds ratio (OR) of Top Three Quartiles Combined (Q2–Q4) Versus the Bottom Quartile (Q1) of SAF for MOF and VFs in the Subset With Available BMD Measurements (*n* = 2654 out of 2853)

	MOF (*n* = 220)		VF (*n* = 183)	
Parameter	OR (95% CI)	*p*	OR (95% CI)	*p*
Q2–Q4 versus Q1 (Ref.)	1.32 (0.94–1.85)	.11	1.71 (1.16–2.53)	**.007**
Adding FN‐BMD	1.30 (0.92–1.82)	.14	1.70 (1.12–2.41)	**.009**
Adding LS‐BMD	1.30 (0.92–1.83)	.14	1.73 (1.17–2.56)	**.007**

Values are shown as ORs (95% CI) per arbitrary unit (A.U.). SAF groups were based on age‐adjusted SAF quartiles. Bold values are significant at *p* ≤ .05.

A.U. = arbitrary units; FN‐BMD = femoral neck bone mineral density; LS‐BMD = lumbar spine bone mineral density; MOF = major osteoporotic fracture; SAF = skin autofluorescence; VF = vertebral fracture.

### Sensitivity analysis

Subgroup analysis was performed in predefined strata of T2DM and non‐T2DM and if the *p* value of interaction term was ≤ .10 for a particular interaction with SAF. First, the association of SAF with MOFs in subjects with T2DM show the same nonlinear trend and higher odds (*p* = n.s. [nonsignificant]) than in subjects without T2DM, but no such trend was observed for the association between SAF and VFs in those with T2DM (Table 4, Supplemental Table 2). Second, there was a statistically significant effect modification in the relationship of SAF and MOFs by sex and smoking (*p* value of interaction < .001 and = .01, respectively), but not for VFs. After stratification by sex, there was a statistically significant association only in females with OR 1.63 (95% CI, 1.11–2.40; *p* = .01) versus males with OR 1.02 (95% CI, 0.55–1.91; *p* = .95) for the association between SAF and MOFs (Table 4). After stratification by smoking categories, there was a significant association observed only in never smokers but not in current or past smokers for the association between SAF and MOFs with OR 2.53 (95% CI, 1.40–2.57; *p* = .002) versus OR 0.75 (95% CI, 0.25–2.23; *p* = .61) and OR 1.05 (95% CI, 0.68–1.62; *p* = .82), respectively (Supplemental Table 3).

**Table 4:** Odds ratio (OR) of Top Three Quartiles Combined (Q2–Q4) Versus the First (Lowest) Quartile (Q1) of SAF for MOF and VFs in Non‐T2DM, T2DM, Females, and Males

	MOFs Q2–Q4 versus Q1 (Ref.)		VFs Q2–Q4 versus Q1 (Ref.)	
Fully adjusted models	ORs (95% CI)	*p*	OR (95% CI)	*p*
Non‐T2DM	1.40 (0.99–1.97)	**.056**	1.52 (0.91–2.53)	.11
T2DM	2.23 (0.64–7.77)	.21	0.57 (0.15–2.27)	.43
Females	1.63 (1.11–2.40)	**.01**	1.48 (0.91–2.41)	.11
Males	1.02 (0.55–1.91)	.95	2.15 (1.14–4.04)	**.02**

Values are OR (95% CI) per arbitrary unit (A.U.). SAF groups were based on age‐adjusted SAF quartiles. Value of *p* for interaction: for MOFs: SAF*DM = 0.39, SAF*sex < .001; for VFs: SAF*DM = .32, SAF*sex = .24.

A.U. = arbitrary units; CI = confidence interval; MOF = major osteoporotic fracture; OR = odds ratio; Q = quartile; Ref. = reference; SAF = skin autofluorescence; T2DM = type 2 diabetes mellitus; VF = vertebral fracture.

## Discussion

In this population‐based study we showed that SAF, a novel non‐invasive biomarker of skin AGEs, was nonlinearly associated with two types of prevalent fractures, namely MOFs and VFs, in the general population. There was evidence for the presence of a threshold value of SAF, below which a lower prevalence of fractures was seen. Importantly, the association between SAF and fractures was independent of BMD. Indeed, FN‐BMD and LS‐BMD showed no association with SAF in the entire cohort, but there was a significant interaction term with sex with a weak but significant inverse association of SAF with FN‐BMD only in females.

A nonlinear relationship between skin AGEs and MOFs including hip fractures was found. Higher serum CML, a frequently studied AGE, was related to higher risk of incident hip fractures in two longitudinal cohort‐studies in elderly subjects.^(^ ^21^, ^41^ ^)^ In one of these studies, in 3384 community‐dwelling men aged 70 to 89 years, a nonlinear relation was found of CML with incident hip fractures, which is similar to our findings of a nonlinear relationship of SAF with prevalent hip fractures.^(^ ^41^ ^)^ Compared to the other two cohorts with a longitudinal design, our subjects were relatively younger with fewer prevalent hip fractures, but we still observed the same trend (Table 5). Urinary pentosidine was associated with incident long‐bone and vertebral fractures in a linear fashion in Japanese subjects (*n* = 765), but no association was found in French (*n* = 396) postmenopausal women.^(^ ^20^, ^42^ ^)^ It should be noted that the serum AGEs used in these studies have different properties and may influence bone through slightly different mechanisms. For example, pentosidine mainly influences collagen crosslinking,^(43^ ^)^ whereas CML is a non‐crosslinking adduct but a potent activator of receptor for AGEs (RAGE).^(^ ^44^, ^45^ ^)^ SAF measured by the AGE Reader has a moderately strong correlation to both pentosidine (*r* = 0.55, *p* < .001) and CML (*r* = 0.55, *p* < .001) in skin^(^ ^26^ ^)^ and may thus take both crosslinking and non‐crosslinking AGEs into account.

**Table 5:** Comparison of Three Studies Including Ours Reporting Measured AGEs and Their Association to Hip Fractures

	Barzilay and colleagues^(^ ^21^ ^)^		Lamb and colleagues^(^ ^41^ ^)^		This study	
Participants	3373		3384		2853	
Males (%)	39.8		100		43	
Age (years)	78 (68–102)		76.3 (74.2–79)		74.1 (66.9–81)	
Data analysis	Longitudinal		Longitudinal		Cross‐sectional	
Mean follow‐up	9.2 years		8–11 years		NA	
AGEs measured	CML		CML		Skin autofluorescence	
Technique used to measure AGEs	ELISA		ELISA		AGE reader	
Estimates of hip fractures per quartile	Fractures per quartile	Hazard ratio	Fractures per quartile	Hazard ratio	Fractures per quartile	Odds ratio
Q1	69	0.94	31	Ref.	5	Ref.
Q2	94	1.34	30	1.13	9	2.08
Q3	81	1.18	17	0.49	6	1.33
Q4	104	1.69	28	0.70	8	1.40

CML = Serum carboxymethyllysine; ELISA = enzyme‐linked immunosorbent assay; NA = not available; Q = quartile; Ref = reference.

We also found evidence of presence of a SAF threshold for their association with VFs. AGEs and vertebral fracture risk has been studied primarily with pentosidine in humans. Shiraki and colleagues^(^ ^46^ ^)^ found the highest quartile of urinary pentosidine to be significantly associated with incident vertebral fracture rate in older females without T2DM, which is in line with what we found in our population without T2DM. A study in young female mice fed with a high‐AGE diet for 6 months showed deterioration of vertebral microarchitecture and a decrease in fracture resistance.^(^ ^47^ ^)^ in vitro glycation studies, on biopsy specimens from both living donors and cadavers, showed that cancellous bone is more susceptible to non‐enzymatic glycation than cortical bone.^(^ ^48^, ^49^ ^)^ The more efficient glycation of bone matrix proteins in cancellous bone is due to higher surface area to volume ratio, which could have facilitated better accessibility of the sugars to the matrix proteins. This could suggest that especially vertebral fracture risk might be increased with increasing levels of AGEs in persons with increased serum glucose levels such as in subjects with T2DM.

In contrast, we observed no associations and not even a trend between SAF and VFs in subjects with T2DM, although others did. Higher serum pentosidine has been associated with prevalent vertebral fractures in elderly T2DM subjects.^(^ ^18^, ^19^ ^)^ Our study differed from these studies in several aspects, namely: (i) lower prevalence of VFs (7% versus 30%) in our diabetics; and (ii) inclusion of participants with T2DM from general population in our cohort versus those referred to hospital outpatient clinics, implying more severe disease in the latter patients. We recently reported in the RS that subjects with T2DM and VFs have a higher mortality compared to those without T2DM and VFs.^(^ ^50^ ^)^ Hence, the absence of a positive association of SAF with VFs in subjects with T2DM in our study might be explained by selective survival apart from low power. A third difference is the use of skin AGEs in our study versus serum AGEs, where skin AGEs may more accurately represent the true tissue burden of AGEs, whereas serum AGEs are more likely to fluctuate over time. Prospective analyses, preferably using skin and serum AGEs simultaneously are needed to further explore these discrepancies.

Various circulating AGEs have been measured with different techniques in the past, which makes the comparison among those studies difficult.^(^ ^51^ ^)^ Furthermore, the amount of circulating AGEs is influenced by dietary AGEs intake and renal clearance.^(^ ^52^ ^)^ We cannot be certain that skin AGEs are a good reflection of bone AGEs, and bone turnover in the elderly might be much higher than skin turnover. There is a lack of good studies comparing skin and bone AGEs at the same time because assessment of AGE levels in bone requires invasive measures.^(^ ^53^ ^)^ Moreover, a recent study in CKD rats (*n* = 56) treated with an AGE breaker, alagebrium, showed reduced total bone AGE content but no difference in bone pentosidine levels, which again points to the heterogeneity in AGEs and limitations of measuring one AGE as a representative of whole group.^(^ ^54^ ^)^

In sex‐stratified analysis for fractures, there was an association of SAF with MOFs only in women but not in men, but no such difference for VFs. Notably in our cohort, the prevalence of MOFs in women is four times higher than in men, which may partly explain this difference. We also found a weak inverse association between SAF and FN‐BMD only in women independent of potential confounders. Other studies also failed to show a strong relation between AGEs and BMD or its surrogates.^(^ ^21^, ^23^, ^29^ ^)^ We suggest that potential sex‐differences between AGEs accumulation and bone strength measures should be studied in prospective studies within well‐powered cohorts. After stratified analysis in current, past, and never smokers, SAF was associated with MOFs only in never smokers, although smoking has been identified as a risk factor for higher skin AGEs accumulation,^(^ ^40^ ^)^ which was also observed in our cohort (data not shown). The fact that we did not find an association between SAF and MOFs in smokers might be related to selective survival and the fact that smoking may increase fracture risk through multiple other mechanisms than AGEs alone.^(^ ^55^ ^)^

There are various mechanisms through which AGEs might deteriorate bone quality and increase fracture predisposition. in vitro studies have demonstrated that binding of AGEs to RAGE inhibits differentiation and enhance apoptosis of osteoblasts.^(^ ^56^ ^)^ A study using osteocyte‐like cells found that AGEs stimulate the expression of sclerostin and inhibit the expression of receptor activator of NF‐κB ligand (RANKL), suggesting a state of low bone turnover.^(^ ^57^ ^)^ AGE‐modification of collagen fibers decreases their degradability by the matrix metalloproteinases either by masking the cleavage site or by preventing unfolding of collagen molecules due to an extra AGEs crosslink.^(^ ^58^ ^)^ AGEs also alter the biomechanical properties of bone tissue by increasing non‐enzymatic crosslinking of collagen fibers, which results in poor bone quality.^(^ ^12^, ^13^ ^)^ In summary, our data primarily suggest that the influence of AGEs on fracture risk is not mediated by low BMD.

In contrast to our expectations, SAF was not associated with TBS, a recently developed 2D textural imaging technique related to bone microarchitecture and derived from DXA images. TBS has been shown by several but not all studies to predict fracture risk largely independent of BMD.^(^ ^59^, ^60^, ^61^ ^)^ Our results suggest that AGEs reflect the material properties of bone independent of TBS (reflecting microarchitecture). AGEs and TBS have been studied together only in one cross‐sectional study (*n* = 112) that found an inverse association between urinary pentosidine and TBS in subjects with T2DM but not in those without T2DM.^(^ ^62^ ^)^ Because both AGEs and TBS have been closely related to bone quality independently of BMD, more studies are needed on the relation between AGEs, TBS, and bone quality.

There are several limitations of our study. As mentioned, AGE levels in skin may not adequately reflect AGEs in bone, although human cadaver studies have shown that the amount of pentosidine per milligram of collagen in both skin and bone were quite close to each other when compared to other tissues.^(^ ^29^ ^)^ Another limitation is that a few MOFs and VFs have occurred quite some time before SAF was measured, which may have influenced the true association with fractures. However, it has been demonstrated that protein turnover is a major determinant of AGEs accumulation in tissues where skin collagen has a long half‐life of 14.8 years.^(^ ^63^ ^)^ This may point toward relatively stable values of skin AGEs over a longer period of time, although we cannot be sure that the half‐life of AGEs on collagen in bone is similar to that of skin. Another limitation is that no serum AGEs were available for comparison with SAF. Although many AGE moieties exist with a diverse range of characteristics, the AGE Reader is only able to detect fluorescent AGEs as SAF, which was shown to be positively correlated to nonfluorescent AGEs measured in skin biopsies.^(^ ^27^ ^)^ Because this is an observational study, we are unable to prove causation and a time gap existed between our outcome and exposure, which may have led to an imprecise estimation of the true association. Despite the availability of many confounders we cannot exclude the existence of residual confounding. Also our data are not generalizable to the non‐Dutch, non‐white, or younger population.

In conclusion, we found a positive association between skin AGEs measured as SAF using the AGE Reader and prevalence of both VFs and MOFs with evidence for a nonlinear relation and a threshold effect. SAF was weakly and negatively associated with BMD in women only and BMD did not explain the observed associations with fractures. Further research with a longitudinal study design is needed to assess the potential use of SAF in prediction of fractures both in subjects with and without T2DM.

## DISCLOSURES

KW, JC, FK, KT, BCE, AGU and FR declare no conflicts of interest. MCZ: Declares having received honoraria in the past for lectures or advice from Alexion, Amgen, Eli Lilly, Kyowa Kirin, Shire and UCB, unrelated to the current work.

## Supporting information


**Supplemental Table 1** (a) and (b). Comparison of logistic regression models including linear term, SAF versus polynomial term, SAF^2^ for both major osteoporotic (MOFs) and vertebral fractures (VFs).
**Supplemental Table 2** Demographic and clinical characteristics of the study participants with skin autofluorescence (SAF) measurements
**Supplemental Table 3** Odds ratio of MOFs and VFs expressed by age‐adjusted SAF quartile in T2DM, non‐T2DM.
**Supplemental Table 4** Effect modification of smoking status on the association between MOFs and SAF as binary variable (Q2‐4 combined vs. Q1.)
**Supplemental Figure 1** Flowchart of participant inclusion from the Rotterdam Study
