# Supplementary material for: Skin Autofluorescence, a Noninvasive Biomarker for Advanced Glycation End‐Products, Is Associated With Prevalent Vertebral and Major Osteoporotic Fractures: The Rotterdam Study
Source: J Bone Miner Res. 2020 Jun 22;35(10):1904–13. doi: 10.1002/jbmr.4096 (PMC7687120; doi:10.1002/jbmr.4096)
Supplement: Supplementary file 1 — Supplemental Table 1 (a) and (b). Comparison of logistic regression models including linear term, SAF versus polynomial term, SAF2 for both major osteoporotic (MOFs) and vertebral fractures (VFs). Supplemental Table 2 Demographic and clinical characteristics of the study participants with skin autofluorescence (SAF) measurements Supplemental Table 3 Odds ratio of MOFs and VFs expressed by age‐adjusted SAF quartile in T2DM, non‐T2DM. Supplemental Table 4 Effect modification of smoking status on the association between MOFs and SAF as binary variable (Q2‐4 combined vs. Q1.) Supplemental Figure 1 Flowchart of participant inclusion from the Rotterdam Study [file JBMR-35-1904-s001.doc]

# **Supplemental data**

**Supplemental Table 1 (a) and (b).** Comparison of logistic regression models including linear term, SAF versus polynomial term, SAF^2^ for both major osteoporotic (MOFs) and vertebral fractures (VFs).

| **a)** |  | **B** | **SE** | **Wald** | **Sig** | **Exp (B)** | **95% CI for Exp (B)** | **-2 Log likelihood (-2LL) ---Akaike information criterion (AIC)** |
| --- | --- | --- | --- | --- | --- | --- | --- | --- |
| Model 1 | SAF | .047 | .073 | .412 | 0.52 | 1.05 | 0.91 - 1.21 | 1515.225 --- 1672.84 |
| Model 2 | SAF | .159 | .09 | 3.22 | **0.07** | 1.17 | 0.99 – 1.39 | 1505.609 --- 1664.46 |
|  | SAF^2^ | -.170 | .06 | 8.03 | **0.005** | .84 | 0.75 - 0.95 |  |

Predicting MOFs from SAF and SAF^2^

**Model 1: MOFs ~ SAF + age + sex + smoking + diabetes + BMI+ Creatinine**

**Model 2: MOFs ~ model 1 + SAF**2

| **b)** |  | **B** | **SE** | **Wald** | **Sig** | **Exp (B)** | **95% CI for Exp (B)** | **-2 Log likelihood (-2LL) ---- Akaike information criterion (AIC)** |
| --- | --- | --- | --- | --- | --- | --- | --- | --- |
| Model 1 | SAF | .105 | .078 | 1.789 | 0.18 | 1.11 | 0.95 – 1.29 | 1384.17 ---- 1410.56 |
| Model 2 | SAF | .257 | .102 | 6.38 | **0.01** | 1.293 | 1.06- 1.58 | 1375.02 ---- 1402.74 |
|  | SAF^2^ | -.179 | .065 | 7.53 | **0.006** | .836 | .74- 0.95 |  |

Predicting VFs from SAF and SAF^2^

**Model 1: VFs ~ SAF + age + sex + smoking + diabetes + BMI+ Creatinine**

**Model 2: VFs ~ model 1 + SAF^2^**

**Supplemental Table 2.** Demographic and clinical characteristics of the study participants with skin autofluorescence (SAF) measurements

|  | **Whole Group N=2853** | **T2DM N=389 (14%)** | **Non-T2DM N=2464 (86%)** |  |
| --- | --- | --- | --- | --- |
| Age, years | 74.08 (14.25) | 75.29 (11.88) | 73.83 (14.46) | **0.001** |
| Females (%) | 57 | 51 | 58 | **0.01** |
| SAF. AU | 2.33±.53 | 2.57±.50 | 2.36±.47 | **<0.001** |
| BMI | 26.99 (5.2) | 29.95 (5.8) | 26.7 (4.9) | **<0.001** |
| Smokers, (%)  Current smokers  Ex-smokers  Never smoked | 15  57  30 | 13  57  30 | 16  52  32 | 0.19 |
| FN-BMD, g/cm^2^ | 0.902±.135 | 0.922±.14 | 0.899±.13 | **0.003** |
| FN-BMD T-score | -1.20±.852 | -1.10±.912 | -1.22±.841 | **0.01** |
| LS-BMD, g/cm^2^ | 1.14±.206 | 1.180±.20 | 1.133±.21 | **<0.0001** |
| LS-BMD T-score | -0.395±1.62 | -0.077±1.58 | -0.438±1.62 | **<0.0001** |
| TBS | 1.308 ±.133 | 1.302±.106 | 1.311±.098 | 0.06 |
| MOFs, n(%)  Hip  Clinical spine  Humerus  Wrist | 245 (9%)  28 (1%)  80 (3%)  39 (2%)  123 (6%) | 35 (9%)  3 (1%)  13(3%)  9 (2%)  12(3%) | 210(8.5%)  23 (1%)  67 (3%)  30 (1%)  111 (5%) | 0.76 |
| VFs, n(%) | 193 (7%) | 26 (7%) | 167 (6%) |  |

*SAF, Skin autofluorescence; BMI, Body mass index; FN-BMD, femoral neck bone mineral density; LS-BMD, lumbar spine bone mineral density; TBS, trabecular bone score; MOFs, prevalent major osteoporotic fractures; VFs, prevalent Vertebral fractures; NA, not applicable.*

*Data are presented as mean ±SD, median (interquartile range) and number (%)*

**Supplemental Table 3.** Odds ratio of MOFs and VFs expressed by age-adjusted SAF quartile in T2DM, non-T2DM.

| **Fully adjusted model** | **Non-T2DM** | | | **T2DM** | | |
| --- | --- | --- | --- | --- | --- | --- |
| **SAF Quartiles** | **MOFs**  **N=224** | **Odds ratio (95% CI)** | **p-value** | **MOFs**  **N=37** | **Odds ratio (95% CI)** | **p-value** |
| **Q1** | 52 | Ref. |  | 3 | Ref. |  |
| **Q2** | 60 | 1.50 (.99-2.26) | **0.05** | 11 | 2.96 (.74-11.9) | 0.12 |
| **Q3** | 51 | 1.19 (.77-1.83) | 0.43 | 13 | 2.88 (.76-10.9) | 0.12 |
| **Q4** | 61 | 1.49 (.98-2.27) | **0.06** | 10 | 1.47 (.38-5.75) | 0.58 |
|  | **VFs N=173** | **OR (CI)** |  | **VFs N=27** | **OR (CI)** |  |
| **Q1** | 31 | Ref. |  | 6 | Ref. |  |
| **Q2** | 46 | 1.89 (1.17-3.06) | **0.009** | 5 | .77 (.21-2.82) .69 | 0.69 |
| **Q3** | 46 | 1.85 (1.14-2.99) | **0.01** | 9 | .99 (.31-3.15) .98 | 0.98 |
| **Q4** | 50 | 2.11 (1.31-3.41) | **0.002** | 7 | .56 (.17-1.86) .35 | 0.35 |

*SAF, Skin autofluorescence; MOFs, major osteoporotic fractures; VFs, Vertebral fractures; Q, Quartiles; T2DM, subjects with type 2 diabetes; Non-T2DM, subjects with no type 2 diabetes; ; CI, Confidence interval.*

**Supplementary Table 4.** Effect modification of smoking status on the association between MOFs and SAF as binary variable (Q2-4 combined vs. Q1.)

| **Fully adjusted models** | **Number of MOFs** | **MOFs Q2-4 combined vs. Q1 (ref.)** | |
| --- | --- | --- | --- |
|  |  | ORs (CI) | p-Value |
| **Current Smokers (N=439)** | 22 (5%) | 0.75 (0.25-2.23) | 0.61 |
| **Ex Smokers (N=1511)** | 136 (9%) | 1.05 (0.68-1.62) | 0.82 |
| **Never smokers N=903** | 87 (9.6%) | 2.53 (1.40-4.57) | **0.002** |

*Major osteoporotic fractures, MOFs; Quartiles, Q*

*p-value of interaction SAF*smoking for MOFs = 0.01*

**Supplementary figure 1** Flowchart of participant inclusion from the Rotterdam Study

Total participants with SAF measurement (N=3029)

SAF outliers (n=8)

No informed consent (n=20)

Missing data on BMI, DM status, smoking and eGFR (n=148)

Missing data on femoral neck (FN) and lumbar spine (LS) bone mineral density and trabecular bone score (TBS) (n=199)

Complete data on SAF, fractures and related covariates (N=2853)

Subgroup with complete data on BMD and TBS (N=2654)
